# Digoxin treatment is associated with an increased incidence of breast cancer: a population-based case-control study

**DOI:** 10.1186/bcr2205

**Published:** 2008-12-03

**Authors:** Thomas P Ahern, Timothy L Lash, Henrik T Sørensen, Lars Pedersen

**Affiliations:** 1 Department of Clinical Epidemiology, Aarhus University Hospital, Olof Palmes Alle 43-45, 8200 Aarhus N, Denmark; 2 Department of Epidemiology, Boston University School of Public Health, 715 Albany Street T3E, Boston, MA 02118, USA

## Abstract

**Introduction:**

Laboratory and epidemiologic studies have suggested a modifying effect of cardiac glycosides (for example, digoxin and digitoxin) on cancer risk. We explored the association between digoxin treatment and invasive breast cancer incidence among postmenopausal Danish women.

**Methods:**

We used Danish registries to identify 5,565 postmenopausal women diagnosed with incident invasive breast carcinoma between 1 January 1991 and 31 December 2007, and 55,650 matched population controls. Cardiac glycoside prescriptions were ascertained from county prescription registries. All subjects had at least 2 years of recorded prescription drug and medical history data. We estimated the odds ratio associating digoxin use with breast cancer in conditional logistic regression models adjusted for age, county of residence, and use of anticoagulants, non-steroidal anti-inflammatory drugs (NSAIDs), aspirin, and hormone replacement therapy. We also explored the impact of confounding by indication and detection bias.

**Results:**

Digoxin was the sole cardiac glycoside prescribed to subjects during the study period. There were 324 breast cancer cases (5.8%) and 2,546 controls (4.6%) with a history of digoxin use at least 1 year before their index date (adjusted odds ratio (OR): 1.30; 95% confidence interval: 1.14 to 1.48). The breast cancer OR increased modestly with increasing duration of digoxin exposure (adjusted OR for 7 to 18 years of digoxin use: 1.39; 95% confidence interval: 1.10 to 1.74). The association was robust to adjustment for age, receipt of hormone replacement therapy, coprescribed drugs, and confounding by indication. A comparison of screening mammography rates between cases and controls showed no evidence of detection bias.

**Conclusions:**

Our results suggest that digoxin treatment increases the risk of invasive breast cancer among postmenopausal women.

## Introduction

Cardiac glycosides (CGs) are natural steroid toxins that have been used since the 18th century to treat congestive heart failure (CHF) and atrial fibrillation (AF) [1]. The clinically most prevalent CGs are the *Digitalis*-derived cardenolides digitoxin and digoxin. These compounds exert their pharmacologic effect via inhibition of the Na^+^/K^+ ^ATPase, which indirectly raises intracellular Ca^2+ ^concentration, thus increasing the force of contractility in cardiac myocytes.

In 1979, Stenkvist *et al*. reported an unusual finding in a small cohort of breast cancer patients (n = 142) [2]. Women in the cohort who were taking CGs (mostly digoxin) at the time of their breast cancer diagnosis had tumors with less aggressive phenotypes than breast tumors of women not taking CGs [2]. They later reported a higher recurrence rate among the women not taking CGs after 5 [3] and approximately 22 [4] years of follow-up. These observations suggested a beneficial effect of cardiac glycosides for women with breast tumors. An early mechanistic hypothesis centered on CG interference with estrogen receptor (ER) signaling in tumor cells [2], while current laboratory studies implicate novel signaling pathways mediated by the Na^+^/K^+ ^ATPase [5,6].

Subsequent studies of the association between CG use and breast cancer incidence gave conflicting results. Haux *et al*. compared site-specific cancer incidence rates among digitalis-treated Norwegians patients with expected rates in the general population [7]. Several cancers, including female breast cancer, occurred at higher rates among those treated with digitalis compared with the general population [7]. Also, Friedman reported no association between CG prescription history and breast cancer in a Kaiser-Permanente registry study [8].

Given the continued importance of CG medicines to treat heart disease and the inconsistent results from earlier studies of the association between this therapy and breast cancer occurrence, we examined the association between digoxin treatment and breast tumor incidence rate in a population-based prospective case-control study of postmenopausal Danish women.

## Materials and methods

This study was approved by the Boston University Medical Campus Institutional Review Board and the Danish Registry Board.

### Study population

This study was conducted within the female population of North Jutland and Aarhus Counties, Denmark [9]. We used county hospital registries to ascertain all cases of incident invasive breast cancer diagnosed in women age 55 or older. Ascertainment began on 1 January 1991 in North Jutland County and 1 January 1998 in Aarhus County, and continued until 31 December 2007 [10]. The hospital registries contain data on patients' civil personal registry (CPR) number, date(s) of admission, date(s) of discharge, and up to 20 discharge diagnoses and medical procedures per discharge or outpatient visit. Diagnoses are assigned by the attending physician, and are coded according to the *International Classification of Diseases*, 8th revision (ICD-8, until 1995) and 10th revision (ICD-10, 1995 onwards).

Controls were identified in the Danish Civil Registration System, which has tracked residential address, vital status, and date of emigration for the entire Danish population since 1968 [11]. Controls were selected for each case by risk-set sampling, matching controls to cases on year of birth and county of residence. Within strata of the matching factors, we selected 10 controls at random among those who were alive and without a history of breast cancer on the date of the matched case's diagnosis. This date was the index date for the cases and matched controls.

### Data collection

We used each subject's unique CPR number to link the case-control roster to county prescription databases [12,13], which automatically record all prescriptions filled since 1989 in North Jutland County and 1996 in Aarhus County. The databases encode drugs by the Anatomical Therapeutic Chemical (ATC) classification system [14] and record dates of all prescription fills along with the patient's CPR number. These systems report prescription data to the county databases, as well as to the Danish National Health Service, which refunds a portion of medication costs. Prescriptions are logged in the registries after patients present to a pharmacy and pay their share of the prescription cost. To ensure adequate prescription data history, we excluded cases and controls who had lived in the study counties for less than 2 years after the establishment of electronic prescription registries. We ascertained medical history for cases and controls by extracting major diagnoses preceding index dates from the county hospital registries. We also used these registries to identify all prediagnosis mammography procedures for cases and controls since 2001, the year mammography data began to be systematically recorded.

### Definitions of analytic variables

We identified cases of incident breast cancer in the hospital registries using ICD-8 and ICD-10 codes appropriate to the date ranges of the databases. ICD codes were also used to ascertain comorbid conditions for cases and controls (see full ICD code listing in Table 1).

**Table 1:** Listing of ICD-8 and ICD-10 codes used to ascertain key diagnoses

Diagnosis	ICD-8	ICD-10
Invasive breast carcinoma^a^	174.00 to 174.02; 174.08; 174.09;	C50.0 to C50.6; C50.8; C50.9
Congestive heart failure	427.09; 427.10; 427.11; 427.19; 428.99; 782.49	I50; I11.0; I13.0; I13.2
Atrial fibrillation/flutter^b^	427.93; 427.94	I48
Myocardial infarction	410	I21; I22; I23
Peripheral vascular disease	440; 441; 442; 443; 444; 445	I70; I71; I72; I73; I74; I77
Cerebrovascular disease	430 to 438	I60 to I69; G45; G46
Chronic pulmonary disease	490 to 493; 515 to 518	J40 to J47; J60 to J67; J68.4; J70.1; J70.3; J84.1; J92.0; J96.1; J98.2; J98.3
Mild liver disease	571; 573.01; 573.04	B18; K70.0 to K70.3; K70.9; K71; K73; K74; K76.0
Moderate to severe liver disease	070.00; 070.02; 070.04; 070.06; 070.08; 573.00; 456.00 to 456.09	B15.0; B16.0; B16.2; B19.0; K70.4; K72; K76.6; I85
Diabetes type 1	249.00; 249.06; 249.07; 249.09	E10.0, E10.1; E10.9
Diabetes type 2	250.00; 250.06; 250.07; 250.09	E11.0; E11.1; E11.9
Moderate to severe renal disease	403; 404; 580 to 583; 584; 590.09; 593.19; 753.10 to 753.19; 792	I12; I13; N00 to N05; N07; N11; N14; N17 to N19; Q61
Diabetes with end organ damage (types 1 and 2)	249.01 to 249.05; 249.08; 250.01 to 250.05; 250.08	E10.2 to E10.8; E11.2 to E11.8
Solid tumor	140 to 194	C00 to C75
Lymphoma	200 to 203; 275.59	C81 to C85; C88; C90; C96

^a^The ICD codes for invasive breast carcinoma do not capture *in situ *tumors (for example, intraductal carcinoma); ^b^ICD-8 contained separate codes for atrial fibrillation (427.93) and flutter (427.94). These two diagnoses were combined into a single code in ICD-10 (I48).

ICD, International Classification of Disease.

We ascertained CG prescriptions by extracting all records from the prescription databases with ATC codes beginning with C01A. CGs are available only by prescription in Denmark, and are dispensed at pharmacies equipped with automated electronic reporting systems described in the data collection section. This strategy captured all CG prescriptions in the counties over the study period that were for digoxin exclusively. Digoxin prescriptions were only considered if they occurred at least 1 year before the index date. Digoxin exposure was considered in broad terms as ever exposed (≥ 1 digoxin prescription at least 1 year before the index date) or never exposed (no record of digoxin prescription at least 1 year before the index date), and in finer terms according to the length of time between a woman's first digoxin prescription and her index date.

Confounders were selected *a priori *based upon established breast cancer risk factors that were also likely to influence receipt of digoxin. Age was initially controlled by matching cases to controls on year of birth. We also calculated each subject's exact age on her index date to adjust for residual confounding by age. We additionally considered confounding by coprescription of anticoagulants, non-steroidal anti-inflammatory drugs (NSAIDs), aspirin, and hormone replacement therapy (HRT). Anticoagulants are frequently prescribed for AF, and were associated with lower risk of urogenital cancer [15]. NSAID use has been associated with increased risk of CHF [16], and these drugs have shown protective associations with breast cancer in some studies [17]. Aspirin use, which may be more prevalent among digoxin users, has been associated with reduced breast cancer risk [18], though data are conflicting [17]. We therefore evaluated confounding by low- and high-dose aspirin use. We also evaluated HRT as a confounder because of its contribution to cumulative hormonal exposure and its association with breast cancer risk [19].

Prescriptions for hormone replacement therapy were identified by ATC codes (estrogens: codes starting with either G03C or L02AA; progestin: codes beginning with G03D; combination therapy: codes beginning with either G03F or G03H). Exposure to any of these drugs before the index date was classified as 'ever exposed to HRT' while exposure to none of them was classified as 'never exposed to HRT'. Similarly, we characterized ever/never exposure to anticoagulants, NSAIDs and aspirin by searching for ATC codes beginning with B01A, M01A, and B01AC06, respectively.

We evaluated confounding by the medical indications for digoxin therapy by defining an alternative reference group of women who were never exposed to digoxin and who had a history of cardiovascular disease (excluding CHF or AF). We hypothesized these reference subjects should be more similar to the digoxin-treated women with regard to cumulative hormonal exposures and lifestyle factors that may modify risk for both heart disease and breast cancer. This reference group also facilitated evaluation of detection bias by allowing comparison of digoxin-exposed women to women with other serious histories who would likely have similar medical usage patterns. We further evaluated detection bias by comparing mammography usage rates between cases and controls. Dates of all mammography procedures among cases and controls were identified in hospital registries using appropriate Danish medical procedure codes. We analyzed mammography usage among women with index dates from 1 January 2006 onward, the period of our study when screening mammography would have been most common in Denmark. For each subject who had undergone mammography before her index date, we identified her most recent procedure and calculated the time elapsed between that procedure and the index date.

### Statistical analysis

We characterized the names, doses, and prescribing frequencies of the various digoxin products used over the study period. We computed the frequency and proportion of cases and controls by digoxin exposure status, prevalent medical conditions, use of other prescription drugs (HRT, anticoagulants, NSAIDs and aspirin), and age on index date.

We calculated the unadjusted odds ratios (ORs) and 95% confidence intervals (CIs) associating digoxin exposure categories with incident breast cancer and used conditional logistic regression to account for the matching factors and to adjust for exact age and past use of HRT, anticoagulants, NSAIDs, and aspirin. Due to the risk-set sampling design, the odds ratio approximates the incidence rate ratio associating digoxin exposure with incident breast cancer [20]. All analyses were performed with SAS version 9 (SAS Institute, Cary, NC, USA).

## Results

### Characteristics of cases and controls

We identified 5,565 cases and 55,650 matched population controls. Among the cases, 324 (5.8%) had ever had a digoxin prescription at least 1 year before her diagnosis date and 2,546 (4.6%) of controls had ever had a digoxin prescription at least 1 year before her index date. The distributions of cases and controls according to age, mammography usage, comorbidity and relevant prescription drug usage are shown in Table 2. By virtue of the matching, cases and controls were identical with respect to age distribution. Cases were somewhat more likely to have CHF, AF, chronic pulmonary disease, or diabetes, and were less likely to have a history of myocardial infarction, than controls. Cases also had more exposure to HRT, anticoagulants and NSAIDs than controls. As expected, mammography usage was substantially higher for cases than for controls in the year preceding the index date (81% vs 1.6%, respectively). However, usage was similar for cases and controls in time periods more distant from index dates.

**Table 2:** Characteristics of the study sample

Variable	Cases (n = 5,565)	Controls (n = 55,650)
Age on index date (years):		
55 to 64	2,116 (38)	21,160 (38)
65 to 74	1,800 (32)	18,000 (32)
75 to 84	1,356 (24)	13,560 (24)
≥ 85	293 (5.3)	2,930 (5.3)

Medical history, n (%):		
Congestive heart failure	160 (2.9)	1,337 (2.4)
Atrial fibrillation/flutter	224 (4.0)	1,819 (3.3)
Prediagnosis mammography^a^		
< 1 year:	417 (81)	84 (1.6)
1 to < 2 years:	3 (0.6)	84 (1.6)
2 to < 3 years:	9 (1.7)	84 (1.6)
≥ 3 years:	17 (3.3)	130 (2.5)
Myocardial infarction	123 (2.2)	1,492 (2.7)
Chronic pulmonary disease	334 (6.0)	3,125 (5.6)
Peripheral vascular disease	167 (3.0)	1,563 (2.8)
Cerebrovascular disease	275 (4.9)	2,842 (5.1)
Lymphoma	12 (0.2)	155 (0.3)
Other solid tumor	0	0
Liver disease	44 (0.8)	403 (0.7)
Diabetes (type I or II)	215 (3.9)	1,706 (3.1)
Diabetes with end-organ complication	85 (1.5)	591 (1.1)
Renal disease	35 (0.6)	446 (0.8)

Other drug exposures, n (%):		
Hormone replacement therapy	2,062 (37)	17,582 (32)
Anticoagulants	231 (4.2)	2,109 (3.8)
NSAIDs	3,106 (56)	29,964 (54)
Aspirin, low-dose (< 150 mg)	205 (3.7)	2,004 (3.6)
Aspirin, high-dose (≥ 150 mg)	505 (9.1)	4,878 (8.8)

^a^Screening mammography data were only available from 2001 onwards. We restricted the mammography analysis to cases and controls with index dates after 1 January 2006, when screening mammography would have been most common in Denmark. Categories reflect time elapsed between most recent mammogram and index date; proportion denominators are the total number of cases (n = 516) or controls (n = 5,160) in the restricted data set.

NSAID, non-steroidal anti-inflammatory drug.

### Digoxin treatment and incident breast cancer

Table 3 shows all of the cardiac glycoside products recorded in the county prescription registries during the study period. We noted that digoxin was the sole CG used during this period. Approximately 97% of all digoxin prescriptions were for 62.5 μg tablets, indicating very little product heterogeneity among the digoxin-exposed subjects.

**Table 3:** All cardiac glycoside products prescribed to study subjects^a^

Product name	Dose	Fill quantity	No. of prescriptions, (% of total)
Digoxin	62.5 μg/tablet	100 tablets	83,094 (66)
	62.5 μg/tablet	200 tablets	38,188 (31)
	250 μg/tablet	100 tablets	4,047 (3.2)
	50 μg/mL	30 mL	28 (0.02)

^a^Result of searching the prescription database for all Anatomical Therapeutic Chemical (ATC) codes beginning with 'C01A'.

We observed a higher rate of breast cancer among ever-users of digoxin, relative to never users, in both unadjusted and adjusted analyses (adjusted OR: 1.30; 95% CI: 1.14 to 1.48; Table 4). This association persisted in categories of drug exposure duration (1 to 3 years, 4 to 6 years and 7 to 18 years), with a suggested upward trend in the odds ratios with increasing duration of digoxin therapy. When we compared digoxin-exposed women with the alternative reference group of unexposed women with cardiovascular medical histories, we continued to observe an association between digoxin exposure and incident breast cancer (adjusted OR: 1.42; 95% CI: 1.14 to 1.77).

**Table 4:** Associations between digoxin treatment and incident breast cancer

Exposure categories	Cases (n = 5,565)	Controls (n = 55,650)	Unadjusted OR (95% CI)	Adjusted^a ^OR (95% CI)
Ever/never prescribed digoxin:				
Ever user	324	2,546	1.29 (1.14 to 1.45)	1.30 (1.14 to 1.48)
Never user	5,241	53,104	1.0 (Ref)	1.0 (Ref)

Duration of digoxin therapy:^b^				
7 to 18 years	93	694	1.35 (1.10 to 1.69)	1.39 (1.10 to 1.74)
4 to 6 years	103	811	1.29 (1.05 to 1.58)	1.30 (1.05 to 1.61)
1 to 3 years	128	1,041	1.25 (1.03 to 1.50)	1.25 (1.03 to 1.52)
Never user	5,241	53,104	1.0 (Ref)	1.0 (Ref)

Ever/never prescribed digoxin (alternate reference group):	(n = 732)	(n = 7,086)		
Ever user	324	2,546	1.42 (1.21 to 1.65)	1.42 (1.14 to 1.77)
Never user^c^	408	4,540	1.0 (Ref)	1.0 (Ref)

^a^Adjusted for age (continuous), county of residence (categorical), and past receipt of hormone replacement therapy, anticoagulants, high- and low-dose aspirin, and non-steroidal anti-inflammatory drugs (NSAIDs) (ever/never); ^b^years elapsed between first digoxin prescription and index date (approximate tertiles of the distribution); ^c^the alternate reference group is additionally defined by a history of myocardial infarction, peripheral vascular disease, cerebrovascular disease, or any combination thereof. See text for rationale.

CI, confidence interval; OR, odds ratio.

## Discussion

Our results suggest there may be a causal association between digoxin treatment and incident breast cancer in postmenopausal women. These findings were robust to adjustment for key confounders, confounding by indication, and medical detection bias.

Interestingly, results from a case-control study by Stenkvist *et al*. agree with our present findings. The investigators compared the CG exposure history of the breast cancer cases from their original report [2] to the exposure history of age-matched controls from the general population [21]. The authors concluded that CGs had no influence on breast cancer incidence, due to a non-significant chi-squared test for independence (p = 0.25). The data from the published crosstabulation in fact yield an OR of 1.39, with a 95% CI of 0.79 to 2.45. While the interval is somewhat wide, the OR is near to our result and consistent with a causal association between CG use and incident breast cancer.

Other previous research is consistent with our results [7,22]. Haux and colleagues observed an elevated breast cancer rate (standardized incidence ratio (SIR): 1.25; 95% CI: 0.95 to 1.62) among mostly postmenopausal digitoxin users, compared with the rate in the general population [7]. The authors also observed elevated SIR for several other cancer sites [7]. Friedman reported results from a Kaiser Permanente cohort study of carcinogenic effects of prescription drugs, which showed no statistically significant association between digitalis treatment and breast cancer incidence. However, the SIR for this association was 1.2 – similar to the result of our study. Ewertz *et al*. found a positive association between digoxin usage and incident male breast cancer (OR for = 5 years of digoxin use: 2.0; 95% CI: 0.9 to 4.4) [22]. Together these results argue against ER antagonism by digitalis glycosides. Our results are more consistent with an ER agonist property of digoxin, though some *in vitro *ER binding studies do not support this notion [23,24].

Recent laboratory findings implicate the Na^+^/K^+ ^ATPase in a variety of signal transduction pathways, with end effects in cell adhesion, survival, and proliferation [25]. Several *in vitro *studies point toward a downstream antiproliferative effect of CGs but others leave open the possibility of cancer-promoting endpoints [26]. The interaction of cardiac glycosides with the Na^+^/K^+ ^ATPase and the consequential effects appear to be highly dependent on the specific CG compound and the subunit makeup of the receiving ATPase [6,26,27]. Therefore it would not be surprising to observe inconsistent responses of different human tissue types to the diverse cardiac glycosides. Some of these ligand-, receptor-, and tissue-specific responses may plausibly result in breast tumorigenesis *in vivo*, consistent with our findings. With this study, we have isolated the association between a single cardiac glycoside, digoxin, and breast cancer incidence in a virtually unselected population of postmenopausal women.

### Strengths and limitations

The main strengths of this study are its large size, use of high-validity registry data to ascertain diagnoses, use of prospectively-recorded exposure information, and lack of selection in enumerating cases and controls.

Our study design minimized the threat of selection bias, which can create the illusion of an exposure-disease association when, in fact, none exists [28]. We had only one subject exclusion criterion, and controls were selected completely at random within strata of the matching factors. Since no subject was required to give their consent to participate, no self-selection mechanism could have influenced our results.

Our results are subject to distortion by residual confounding and misclassification of exposure and outcome. We took measures to address confounding by age, past exposure to other prescription drugs, and the medical indications for digoxin prescription. We saw little change in the unadjusted association after accounting for these factors. Digoxin is ordinarily prescribed at an age when most women no longer bear children, so it is unlikely that digoxin exposure is strongly associated with the well-characterized reproductive factors that affect breast cancer risk [29]. We therefore do not expect substantial residual confounding. It is unlikely that use of other prescription drugs could bias our results, since antibiotics, antihypertensives, statins, and antidepressants do not appear to modify breast cancer risk [17]. Use of the alternative reference group resulted in a modest increase in the estimated odds ratio; this result implies that confounding by indication actually served to attenuate the original association. Furthermore, detection bias is not likely to account for the observed association, since women with other cardiovascular diseases would have similar medical usage to women treated with CG. In the whole study population, we saw no material difference in mammography usage rates between cases and controls in time periods distant from index dates, which further argues against detection bias.

We were not able to adjust directly for body mass index (BMI), which is associated with both cardiovascular disease (CVD) and breast cancer [30]. However our alternative reference group likely controlled in part for BMI due to the association of BMI with CVD [31]. Since the effect of adjustment via this reference group was to move the odds ratio estimate away from the null, it is unlikely that unmeasured confounding by BMI could account for our positive result.

Our characterization of digoxin exposure was informed only by the number and strength of prescriptions filled by study participants; the prescription registry data did not permit calculation of actual daily doses taken by exposed subjects. Because prescription records were generated automatically before breast cancer diagnoses, we expect any exposure classification error to be non-differential in nature. We are not aware of published validation data on the classification of incident breast cancer in the hospital discharge registries. However, breast cancer diagnoses were recorded without express knowledge of exposure, so outcome misclassification is also expected to be non-differential. Since non-differential classification errors are expected to attenuate results, exposure and outcome misclassification cannot plausibly account for our positive association [28].

## Conclusion

We observed a modestly increased rate of breast cancer among postmenopausal women with any history of digoxin use, compared with women with no such use, after adjustment for age, use of other prescription drugs, and cardiovascular indications. The associations persisted in long-term exposure categories. While a number of laboratory studies of cardiac glycosides and female breast cancer have suggested protective effects, our results suggest that one specific cardiac glycoside, digoxin, moderately increases the incidence rate of breast cancer. This finding agrees with results from past studies; [7,8,21] the importance of which were likely masked by large standard errors of the association measures.

## Abbreviations

AF: atrial fibrillation; BMI: body mass index; CG: cardiac glycoside; CHF: congestive heart failure; CI: confidence interval; CVD: cardiovascular disease; ER: estrogen receptor; HRT: hormone replacement therapy; ICD: International Classification of Diseases; OR: odds ratio; SIR: standardized incidence ratio.

## Competing interests

HTS reports receiving no fees, honoraria, grants or consultancies. The Department of Clinical Epidemiology is, however, involved in studies with funding from various pharmaceutical companies (Amgen, Pfizer, Glaxo and Centocor) as research grants to (and administered by) Aarhus University. None of these studies have relation to the present study. TPA, LAP and TLL declare no conflicts of interest.

## Authors' contributions

HTS, TPA and TLL conceived the study idea. TPA, TLL and HTS designed the study. HTS and LAP collected the data. TPA performed all data analyses, reviewed the literature and wrote the first draft of the manuscript. All authors edited the manuscript.
